# Dataset on demographic and Socio-economic triggers of informal settlements: a case study from the peri-urban areas of Woldia

**DOI:** 10.1016/j.dib.2020.105667

**Published:** 2020-05-08

**Authors:** Fentaw Baye, Fisseha Wegayehu, Solomon Mulugeta

**Affiliations:** a Ethiopian Institute of Architecture, Building Construction and City Development (EiABC), Addis Ababa University, P.O. Box 518, Addis Ababa, Ethiopia; b Faculty of Social Science, Department of Geography and Environmental Studies, Addis Ababa University, P.O. Box 1176, Addis Ababa, Ethiopia

**Keywords:** Housing condition, Income, Informal settlement, Land market, Woldia

## Abstract

The data collection strategy involved the use of multiple methods. While Primary source of data were collected through the use of structured and semi-structured interviews, focus group discussions and a questionnaire household survey; secondary data were gathered from published and unpublished materials and land related legal and policy documents. Primary data were gathered through house to house survey directly administered to a random sample of 244 household heads. Besides to the household survey, primary data were collected via in-depth interviews, and focus group discussion.

Interviews were conducted with land brokers, land speculators, key informants and governmental officials. Focus group discussion was executed in two different sessions: one from kebele 04 and the other from the municipality. A total of 87 people from government officials, land brokers and speculators and key informants were interviewed. Secondary data were collected from available documents and land-related legal and policy documents.

The data collection process is followed by a detailed qualitative and quantitative data analysis. The qualitative analysis part includes analysis of data obtained from interviews and focus group discussions. However, statistical analysis includes descriptive statistics such as cross tabulation, percentage and correlation were employed using IBM SPSS 20.

Informal settlement continues to be a challenge in the contemporary urbanization in Ethiopia. Thus, these dataset have important implications for urban land policy both at local, national and wider audience beyond Ethiopia to reconsider urban informality. The data of this manuscript is associated with the publication [10.1016/j.landusepol.2020.104573].

Specifications Table

**Table:** 

Subject	Urban and regional planning
Specific subject area	Urban informal settlement
Type of data	Data tables and figures in Word files
How data were acquired	To do the present research, 244 peri-urban households were selected for questionnaire administration besides to interviews, and focus group discussion as a source of data. After completing the questionnaires, the results were analysed using the statistical SPSS software version 21. Data obtained from focus group discussion and interview were transcribed to the suitability of the study. Survey questionnaires are indicated in the supplementary material of this article.
Data format	Raw and refined data
Parameters for data collection	The primary parameters for data collection include socio-economic and demographic related criteria based on their relevance and degree of distinguishability of informal settlements to the topic being discussed.
Description of data collection	Household survey was conducted in the peri-urban areas of Woldia: *Adengur, Wassie, Ariro, Foot of Gebrael, Commanda Teba, Kore* and *Tinfaz*. The survey was executed by means of a questionnaire administered to 244 sample peri-urban households, and structured and semi-structured interviews as well as focus group discussions with 87 individuals from governmental officials, land brokers, land speculators, key informants. A total of 331 participants in six categories were participated. Besides, available manuals and land related legal and policy documents were reviewed [2]. Questionnaires for collecting the data are included in the supplementary material in this article
Data source location	Woldia town, Amhara National Regional State Ethiopia
Data accessibility	With the article
Related research article	Baye, F., Wegayehu, F., & Mulugeta, S. (2020). Drivers of informal settlements at the peri-urban areas of Woldia : Assessment on the demographic and socio-economic trigger factors ⋆. *Land Use Policy, 95*, 1–11. https://doi.org/10.1016/j.landusepol.2020.104573

## Value of the data

• Data can be used to supply the local governments with the necessary information they need to make informed decisions
• The data can provide new insights to stakeholders to manage, update and explore alternative housing delivery methods in order to speed up the overall accessing process at a point in time.
• The data can provide useful information to bring anyone who is interested to realize the challenges of urban areas in Ethiopia and the issue of informal settlement on the ground in particular [2].

## 1 Data

**Fig. 1:**
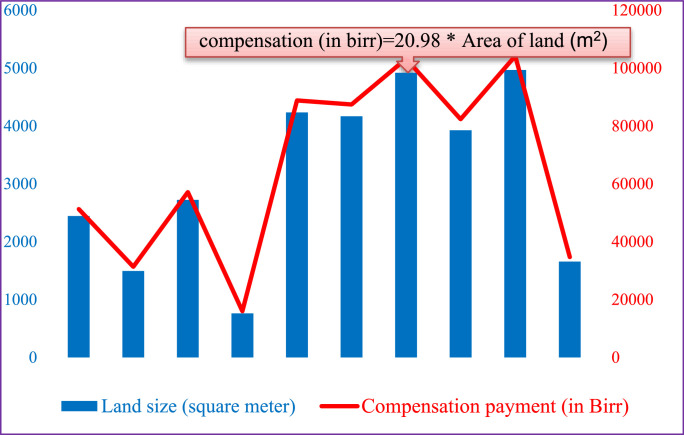
Correlation between land size and compensation payment

The present dataset is presented in tables and figures. These datasets are description of socio-economic and demographic triggers of informal settlers at the peri-urban areas of Woldia. The data were collected using questionnaires (Table 1) and in-depth interviews for the formal and informal land markets (Table 2 and Fig. 1). Sample respondents at the informally occupied areas, in addition to other questions, were requested to answer regarding their socio-economic and demographic as well as housing characteristics [1]. The overall responses are presented in Table 1 and Fig. 1.

**Table 1:** Demographic and socio-economic of sample respondents

Characteristics	Number	%
**Sex**		
Male	90	36.9
Female	154	63.1
**Marital status**		
Married	161	66
Never married	31	12.7
Divorced	20	8.2
Separated	7	2.9
Widow/widower	25	10.2
**Educational characteristics**		
Illiterate	50	20.5
Read and write only	23	9.4
Primary (Grade 1-8)	44	18.0
Secondary (grade 9-12)	67	27.5
Certificate	4	1.6
Diploma and above	56	23.0
**Average monthly income**		
≤ 600	52	21.3
601-1650	75	30.7
1651-3200	56	23.0
3201-5250	39	16.0
5251-7800	15	6.1
7801-10900	4	1.6
≥ 10901	3	1.2
**Housing types**		
Detached	210	86.1
Connected multifamily	34	13.9
**Number of rooms**		
One	19	7.8
Two	45	18.4
Three	73	29.9
Four	107	43.9
**Source of income to build the house**		
Self/savings	155	63.5
Informal borrowing without interest	8	3.3
Informal money lender with interest	13	5.3
Formal loan with collateral	14	5.4
Other	54	22.4
**Main uses of the house**		
Residential	235	96.3
Both residential and commercial	8	3.3
Other	1	0.4
**Main construction materials of walls**		
Mud and wood	214	87.7
Stone and brick	6	2.5
Corrugated ion	2	0.8
Flattened tin cans	20	8.2
Others	2	0.8
**Main construction materials of roof**		
Concrete	5	2.0
Asbestos sheet	4	1.6
Corrugated iron	235	96.3
Thatch	0	0
Other	0	0
**Main construction materials of floor**		
Soil/earthen	144	59.0
Tiles/marble	29	11.9
Concrete	65	26.6
Wood	1	0.4
Other	5	2.0
**Main construction materials of ceiling**		
*Cloth/Abujed*	38	15.5
*Chipudi*	27	11.1
Textiles	110	45.1
Other	49	20.1
No ceiling	20	8.2
**Means of getting the land**		
Gift	27	11.1
Lease	43	17.6
Inheritance	22	9.0
Freely squatting	17	7.0
Others such as buying	135	55.3

**Table 2:** Time needed for saving in order to afford housing plots in the formal and informal markets

Thresholds	Formal market		Informal market	
	Minimum	Maximum	Minimum	Maximum
	150,000birr (US $ 5,265)	1,500,000birr (US $ 52,650)	120,000birr (US $ 4212)	320,000birr (US $ 11,232)
Low income	5 years	53 years	4 years	11 years
Lower middle income	1 year-5 years	13 years-53 years	1 year- 4 years	3years-11 years
Upper middle income	4 months-1 year	6 months-4 years	4 months-1 year	11 months-3 years

Table 1 describes the various socio-economic, demographic and housing conditions of the respondents. Among the demographic/social variables, age, marital status and highest educational attainment have been examined. It also describes the housing conditions of the sample respondents. Accordingly, housing type, number of rooms, main use of the houses and types of construction materials for roof, floor, wall and ceiling have been investigated. Regarding the economic issues, it also describes the average monthly income, the main sources of income to build houses and means of getting land for housing.

Table 2 describes the amount of time/year/needed to save money by household respondents given the prevailing income level of respondents and the housing market price in the peri-urban areas of Woldia both in the formal and informal marketing. To own a residential house on the current market price and prevailing average monthly income of the respondents, the amount of time needed ranges from a minimum of 4 months to 53 years in the formal market and from 4 months to 11 years in the informal market (Table 2).

Fig. 1 describes the correlation between land size and compensation payment made to peri-urban farmers. As the size of the farm land increases, the corresponding compensation payment to peri-urban farmers also increases and vice versa. Being other things constant, there is a positive correlation between farm land size and compensation payments.

## 2 Experimental Design, Materials, and Methods

Prior to starting the actual data collection, field assessment was undertaken on a selected study areas, the respective *kebeles and* departments in the municipality of Woldia. Moreover, since the first draft of the questionnaire was prepared in English, it was translated in to Amharic version, local language of the respondents, to avoid free translation and thus misconception of the questionnaires by the enumerators. In translating the questionnaire, two post graduate students from the department of English language and literature were consulted. To validate whether there exists vagueness, misunderstanding and other weaknesses on the first draft of the questionnaire or not, a pilot test of the first draft was administered upon 4 informal settlers prior to the actual field work. On the basis of the validation, hence, the instruments have further refined.

Thus, primary data were collected via household survey, in-depth interviews, and focus group discussions. Household survey was conducted in *Adendur and Wassie, Ariro and Foot of Gebrael Mountain, Commanda Teba, Kore* and *Tinfaz* [2]. Thus, sample respondents were selected from these areas because footprints of informal settlements were more visible than other places. The household survey was conducted by moving from house to house to 246 households, but two questionnaires have been rejected due to misinformation. For questionnaire administration, 5 enumerators (3 males and 2 females who are grade 12 students) were selected and trained how to approach, ask interviewees, and handle the challenges that may come across during the field work. Besides, there were 5 supervisors (who are teachers from Woldia Preparatory and Higher Education secondary school) in each of the data collection areas.

Moreover, given the unauthorized nature of informal settlements and the question of willingness of sample respondents, the data collectors were purposely selected from peri-urban households where they come from. This is because research participants knew and trusted them, the data collectors, than a strange face during questionnaire administration. Furthermore, official supporting letters for enumerators and supervisors were brought from Woldia town mayor office to make sure that the data collectors and supervisors are legal. Finally, household survey was conducted from the 24^th^ of January 2019 to the 31^th^ of January 2019 during the school holidays/vacations and the first two consecutive weekends (Saturday and Sunday) of February 2019.

In addition to the peri-urban households, data were collected from governmental officials, land brokers, land speculators, key informants, and focus group discussion using structured and semi-structured interviews. Interviews were executed with land brokers (5 in number), land speculators (2 in number), key informants (8 in number) and governmental officials (63 in number) with the researcher. The governmental officials were from municipality, mayor, zone land administration, *kebeles* offices. Focus group discussion was conducted in two different sessions, one from kebele 04 (6 in number) and the other from experts of the municipality (4 in number).

The data obtained from interviews and focus group discussion responses were transcribed and analysed. Moreover, to substantiate the data collected through the instruments mentioned above, available documents and land-related legal and policy were reviewed [2].

Data obtained using interview, and focus group discussion were qualitative in nature. This was because, qualitative research method brings face to face with the real world to be investigated; involves close contact between the researcher and the research participants which are interactive and developmental; allow for emergent issues to be explored; and data which are very detailed, information rich, and expensive [3].

## Appendix Supplementary materials

Image, application 1
