# Supplementary material for: Dataset on demographic and Socio-economic triggers of informal settlements: a case study from the peri-urban areas of Woldia
Source: Data Brief. 2020 May 8;30:105667. doi: 10.1016/j.dib.2020.105667 (PMC7221159; doi:10.1016/j.dib.2020.105667)
Supplement: Supplementary file 1 [file mmc1.docx]

***Questionnaire for settlers/residents/farmers***

***Part I Household information***

1. Site name-------------------------------
2. Code number of the respondent (serial number given to the respondent) ----------
3. Sex of the respondent------------------------------- 1) Male 2) Female
4. Marital status 1) Married 2) Never married 3) Divorced 4) Separated 5) Widow/widower
5. What is the highest educational level you have achieved?

1) Illiterate 2) Read and write (with no formal education) 3) Primary (1-8)

4) High school (9-12) 5) Certificate 6) Diploma and above

1. What is the average monthly income (in Birr) of the household?

1) <600 2) 601-1650 3) 1651-3200 4) 3201-5250 5) 5251-7800

6) 7081-10900 7) ≥10901

**Part II house and housing condition related issues**

**Type of house**

1. Dwelling is in 1) Detached house 2) connected multifamily house

2. How did you fund the purchase or construction of this dwelling?

- 1. Own sources 3) Informal loan / money lender (with interest)
  2. Informal borrowing (with no interest) 4) Formal loan (with collateral) 5) other specify

3. How many rooms do the members of your household occupy, including bedrooms, living rooms and rooms

used for household enterprises? 1) One 2) Two 3) three 4) More than three

1. Function of the constructed house
   1. Own residential 2) Commercial and residence 3) other specify
2. What are the main construction materials of the external ***wall*** of the house?

1) Mud and wood/logs 2) Stone and brick 3) Corrugated iron 4) flattened tin cans

5) Any other/specify

1. What are the main construction materials of ***roof*** of the house?
   1. Concrete 3) Asbestos sheets 4) Corrugated iron 5) tile
   2. thatch 6) Any other/specify
2. Whata are the main construction materials of the ceiling?
   1. *Abujedi*/cloth 2) *Chipudi* 3) Textiles 4) No ceiling 5) Other/specify
3. What are the main construction materials of ***floor*** of the house?
   1. Soil/earthen floor 3) tile 4) concrete
   2. painted( wood) 5) Any other/specify
4. Under what conditions do you occupy the land on which your dwelling is located?
   - - - 1. Family gift 2) leased 3) inheritance 4) unlawful/squatted 5) other/ specify

***Open-ended questionnaires for land brokers/speculators***

- 1. For how many years have you been living in this locality/area?
  2. Did you involve any other income generating activities other than working as a land broker? If any what is that?
  3. What are the most common plot sizes requested by buyers?
  4. How price setting is determined?
  5. What is the price of the plot size? Is there a variation on the cost of the plot of land across different sites such as Ariro, Adengur, and Michael…?
  6. How do you characterize the economic status of people who wants to acquire a plot through informal means?
  7. Who are the main sellers of plots of lands in the peri-urban areas?
  8. Did you observe that the demand for informal land acquiring is changing (i.e. from low income earner to middle and high income earner)?
  9. Did you know the reasons why informal settlers are entering to the informal land market as opposed to the formal land delivery mechanism?

***Open-ended questionnaires for government officials***

- 1. Regardless of many efforts to stop informal/unlawful land occupations in the peri-urban areas, the constructions of these settlements are flourishing alarmingly even today. Who should be blamed on the increasing of unlawful settlements in the town? Why the government failed to do so?
  2. Many people agree that the main causes for unlawful occupation and informal land transaction at the peri-urban areas are misguided policy and weak regulation system, particularly the lease policy and Regulation 721*/2011 and regulation 103/2012*. What is your opinion on this?
  3. Many people criticized that the key shelter problems faced by urban people especially the poor includes access to land, the cost and financing of housing, eligibility requirements, access to information, procedures and regulations. What is the role of the land administration office in this regard?
  4. Is there an informal Housing/settlement Act as an instrument to promote various forms of informal housing development and resolve conflicts and complex issues of property rights that will inevitably arise
  5. To what extent the lease office *(region and city administrations*) *updates the benchmark lease price of urban* land corresponding to the up-to-date market information or in line with inflation?*(every two years(proclamation 721/2011, article, 14-3)?*
  6. The leased land once leased to the leaseholder involved multiple transactions for sale informally which influences the internal revenue of the town. What is your comment on this?
